# A Classification Scheme for Analyzing Mobile Apps Used to Prevent and Manage Disease in Late Life

**DOI:** 10.2196/mhealth.2877

**Published:** 2014-02-17

**Authors:** Aiguo Wang, Ning An, Xin Lu, Hongtu Chen, Changqun Li, Sue Levkoff

**Affiliations:** ^1^Gerontechnology LabHefei University of TechnologyHefeiChina; ^2^Environment and Health Group, IncCambridge, MAUnited States; ^3^Department of PsychiatryBrigham and Women's HospitalHarvard UniversityBoston, MAUnited States; ^4^Global Initiative on Caregiving for the Elderly, Asia CenterHarvard UniversityCambridge, MAUnited States; ^5^College of Social WorkUniversity of South CarolinaColumbia, SCUnited States; ^6^Department of Global Health and Social MedicineHarvard Medical SchoolCambridge, MAUnited States

**Keywords:** mHealth, app, Precede-Proceed Model (PPM), health care process, prevention, management, physical health, mental health

## Abstract

**Background:**

There are several mobile apps that offer tools for disease prevention and management among older adults, and promote health behaviors that could potentially reduce or delay the onset of disease. A classification scheme that categorizes apps could be useful to both older adult app users and app developers.

**Objective:**

The objective of our study was to build and evaluate the effectiveness of a classification scheme that classifies mobile apps available for older adults in the “Health & Fitness” category of the iTunes App Store.

**Methods:**

We constructed a classification scheme for mobile apps according to three dimensions: (1) the Precede-Proceed Model (PPM), which classifies mobile apps in terms of predisposing, enabling, and reinforcing factors for behavior change; (2) health care process, specifically prevention versus management of disease; and (3) health conditions, including physical health and mental health. Content analysis was conducted by the research team on health and fitness apps designed specifically for older adults, as well as those applicable to older adults, released during the months of June and August 2011 and August 2012. Face validity was assessed by a different group of individuals, who were not related to the study. A reliability analysis was conducted to confirm the accuracy of the coding scheme of the sample apps in this study.

**Results:**

After applying sample inclusion and exclusion criteria, a total of 119 apps were included in the study sample, of which 26/119 (21.8%) were released in June 2011, 45/119 (37.8%) in August 2011, and 48/119 (40.3%) in August 2012. Face validity was determined by interviewing 11 people, who agreed that this scheme accurately reflected the nature of this application. The entire study sample was successfully coded, demonstrating satisfactory inter-rater reliability by two independent coders (95.8% initial concordance and 100% concordance after consensus was reached). The apps included in the study sample were more likely to be used for the management of disease than prevention of disease (109/119, 91.6% vs 15/119, 12.6%). More apps contributed to physical health rather than mental health (81/119, 68.1% vs 47/119, 39.5%). Enabling apps (114/119, 95.8%) were more common than reinforcing (20/119, 16.8%) or predisposing apps (10/119, 8.4%).

**Conclusions:**

The findings, including face validity and inter-rater reliability, support the integrity of the proposed classification scheme for categorizing mobile apps for older adults in the “Health and Fitness” category available in the iTunes App Store. Using the proposed classification system, older adult app users would be better positioned to identify apps appropriate for their needs, and app developers would be able to obtain the distributions of available mobile apps for health-related concerns of older adults more easily.

## Introduction

### Background

 According to the United Nations [1], globally increasing life expectancy and decreasing birth rates have created a pervasive phenomenon of population aging, affecting both developing and developed countries. Countries are already experiencing public health challenges due to increased prevalence of chronic diseases, many of which are the result of poor health behaviors, and concomitant economic challenges, associated with increased medical expenditures for disease management and treatment. Projections indicate that by 2050, older adults (ie, individuals aged 65 years and older) will account for 21% of the global population [1]. Given data that suggest older adults consume over two thirds of medical resources [2], further aging of the population is likely to strain governments’ ability to provide care [3,4]. In addition to increasing costs of care, chronic disease also directly affects the quality of life of both elders and their family members [5].

Concurrent with global population aging is the rapid development of mobile technologies that have the potential to improve the quality of life and enhance the independence of older adults. Mobile technologies are promising, as they offer continuous availability from anywhere at any time; offer interactive user interfaces with multimedia capabilities to engage users; require low levels of infrastructure provision, enabling their use in remote areas and providing significant economic benefits to these areas [6]; and offer the possibility of uninterrupted collection of personal health data for positive behavior change. Some examples of mobile technologies include remote monitoring of falls and physiological data collection through smart homes deployed with sensor networks, which enables the collection of data on a variety of health outcomes and has the ability to send the data to appropriate formal health care providers or informal caregivers [7-9]. Apps such as these, which are equipped with location-based services and covered by wire/wireless network signals, offer the potential to conduct pervasive and ubiquitous health interventions, and to reduce the high rates of attrition, which is often found in interventions that rely on participants’ uploading of data [10]. The momentum, at which the volume and range of mobile apps have been steadily expanding, provides a foundation on which to promote the adoption of smartphones among older adults, as well as younger aged individuals, and further to promote the use of mobile technologies to improve the quality of life and enhance the independence of older adults [11,12].

The adoption of mobile technology is beginning to far outpace personal computer (PC)-based ones. Chen pointed out that in 2011, the annual global smartphone shipments reached 488 million, 62.7% growth, more than PC shipments [11]. Allied Business Intelligence (ABI) Research recently released a report predicting that the sport and health mobile app market would hit US$400 million in revenues by 2016, quadrupling the revenues of 2010 [12]. In 2010, there were only 5805 health, fitness, and medical apps available within the Apple App Store [13]. That number reached more than 13,600 by 2012, more than doubling in 2 years. Kahn pointed out that at the end of 2009, 80% of Americans owned a cell phone, a personal digital assistant (PDA) phone, or a smartphone, and that there were a rapidly growing number of people over the age of 65 using smartphones [13]. These trends demonstrate that it is feasible and likely that older adults will use smartphones to increase their knowledge of prevention and self-management of disease, both physical and mental health conditions, as well as to apply the appropriate tools that help with behavior change for healthy lifestyles. To respond to this increasingly large potential market, smartphones are being tailored for older adults with larger displays, easy-operating keyboards, and simple but powerful functions. Mobile apps also present the possibility for family caregivers to take care of their loved ones more efficiently and effectively, for example, by being notified through remote monitoring of a potentially dangerous fall, as well as for professional caregivers, who receive monitoring data in a more timely fashion, which allows for more prompt treatment.

Researchers have conducted a number of studies on how to design and develop useful mobile apps and how to improve user experiences, such as developing apps with low cognitive complexity, motivating the elderly in using apps, and taking vision and hearing impairment into account [14,15]. Moreover, mobile apps specifically for improving quality of life of older adults have been identified and developed [10,15]. For example, Boulos et al described the development of a smartphone app within Enhanced Complete Ambient Assisted Living Experiment (eCAALYX), which is targeted at improving the quality of life and health conditions for older adults. This smartphone app receives inputs from wireless health sensors and location sensors and sends these data to a remote server through the Internet, where they are accessible to professional caregivers, who can respond quickly, thus facilitating the fast response to at risk situations of older adults and improving quality of life [10].

Despite the surging trends in the development of mobile apps to improve health and health care conditions, a gap exists between available apps and the actual demands from users [15]. Because of the lack of comparable market information, this could lead to mobile application developers designing and developing a number of apps with similar functions and characteristics to those already in use, resulting in an imbalance in the availability and distribution of mobile apps. Few studies, however, provide insights regarding current trends and gaps of this increasingly crowded mobile health application market. Developers are not able to quickly make informed decisions on which areas to devote their development efforts, given the lack of state-of-the-art and dynamically varying mobile apps market information. This could potentially lead to wasted time and resources on the part of app developers.

There also exists a gap in the ability of older adults to choose mobile apps that are appropriate to their specific health concerns. For example, some older adults might want an app that provides information on how to manage anxiety or prevent diabetes, as well as an app that identifies reinforcing factors to help them in their health goals.

Our proposed research expands on the work by West et al [8], which categorized mobile apps by the Precede-Proceed Model (PPM) model (predisposing, enabling, and reinforcing factors) [16,17], by providing two additional dimensions: (1) whether the apps are for management of disease and/or prevention and (2) whether they are related to physical and/or mental health. These information have important implications for both older adults seeking apps for specific purposes, as well as for mobile app developers, to help them better understand the distributions of different kinds of mobile apps on the market.

### Precede-Proceed Model

West and colleagues [8] took the important first step in mobile app classification by using both the Health Education Curriculum Analysis Tool (HECAT) and the Precede-Proceed Model (PPM) to analyze the existing health and fitness apps in the iTunes App Store. HECAT’s classification criteria include specific health-related behaviors, such as alcohol consumption, healthy eating, tobacco consumption, and other drugs [18]. Using the HECAT system, West and colleagues concluded that apps related to physical activity and personal wellness were the most common. As to the PPM, they also found that most of apps were classified as predisposing (1776/3336, 53.24%) or enabling (2181/3336, 65.38%), with reinforcing apps being the least common (222/3336, 6.65%) and with very few apps possessing all three factors (62/3336, 1.86%). Conceptually, PPM is a stronger model than HECAT because PPM provides a framework for creating health promotion interventions that are not targeted to any specific health behavior. Nevertheless, PPM lacks the capability to incorporate specific health conditions, to which HECAT pays particular attention, and health care processes, which include prevention and management.

In this paper, we propose a new classification scheme to analyze mobile apps on the market, extending the PPM model to include two important additional dimensions: physical versus mental health and prevention versus management. We believe that this more comprehensive analysis of mobile apps offers several advantages when compared to the PPM model alone. First, since our scheme is built on the PPM model, it still retains the capacity of PPM to classify or analyze mobile applications into predisposing, enabling, and reinforcing categories. Second, our scheme incorporates prevention and management of disease. Since prevention and management of disease have different emphases and are both of great importance in health care processes, it is a natural addition to West’s original classification scheme. Third, while not as detailed as the HECAT system, the proposed scheme does allow classification by physical/mental health. Finally, the proposed scheme allows us to analyze existing mobile apps according to either PPM, health/mental health, or management/prevention separately, as well as to examine the overlap of mobile apps in various categories (eg, mental health-related apps for prevention that provide information on enabling skills).

For testing and comparative purposes, we collected information on mobile apps for older adult health that were released in June and August, 2011 and August 2012 from the “Health & Fitness” category in the Apple iTunes App Store and applied the proposed classification scheme to categorize these apps. Our purpose was to develop a classification scheme to facilitate the selection of mobile apps by older adults so that they can choose most appropriate apps for them (eg, depending on whether they want an app that is predisposing, enabling, or reinforcing, whether they want an app for health or mental health conditions, and whether they are interested in prevention and/or management of disease), as well as to provide mobile app developers a way of obtaining more specific and valued insights into the field of existing mobile apps.

## Methods

### Design

**Figure 1:**
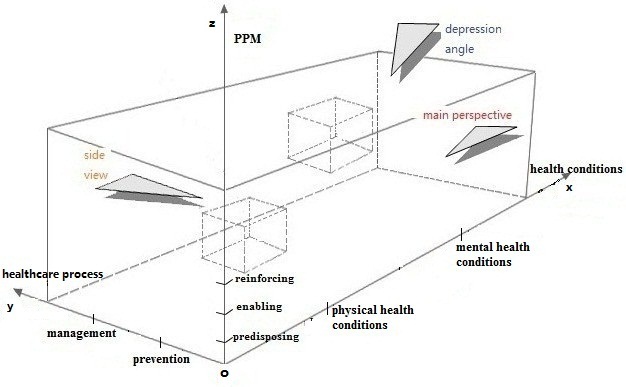
Proposed scheme for apps classification.

After a qualitative content analysis of manufacturers’ description of health and fitness apps was conducted by the research team, we constructed a classification scheme for the classification of mobile apps according to three dimensions: (1) the PPM [16,17], (2) prevention versus management of disease, and (3) physical versus mental health. Figure 1 shows the constructed classification scheme.

The x-axis of the classification scheme represents health conditions, including physical health conditions such as hypertension, diabetes, heart diseases, bronchitis, and stomach flu, as well as mental health conditions such as depression, social phobia, acute stress disorder, anxiety, and schizophrenia. We categorize health conditions into physical health conditions and mental health conditions because this categorization is reflective of the development structure of most health-related apps. Although mental health and physical health commonly impact each other [19], most current apps have their main objective to benefit either mental or physical health exclusively. However, if an app is clearly associated with both physical health and mental health conditions, it is coded in both categories.

The second dimension, the y-axis, represents health care processes. As mobile apps are increasingly playing a major role in facilitating the diffusion of health information, enabling positive behavior change, longitudinal and continuous collection of personal health-related data, remote monitoring and warning, and other health care activities, apps could be regularly and commonly used for the prevention and/or management of disease [20,21]. Prevention tools help individuals manage risks for certain diseases, by, for example, promoting healthy lifestyle behaviors, such as healthy diet and physical activity, which are risk factors for many of the major chronic diseases in late life. Apps for the management of chronic disease place an emphasis on providing individuals with the information, skills, and associated tools to monitor their conditions, to react promptly when a certain type of clinical intervention might be needed to avoid a worsening of symptoms and/or costly hospitalization, as well as to aid older adults in the recovery process [22,23]. Therefore, we choose prevention and management of diseases to be the two components of the y-axis of the scheme. Similar to the physical/mental health dimensions, if an app is clearly associated with both prevention and management (eg, self-monitoring of blood pressure can serve both purposes), it is coded in both categories.

The third dimension, the z-axis, is adapted from the PPM model, which identifies the functions or goals of health-related mobile apps into three categories: predisposing, enabling, and reinforcing. According to the health promotion framework, predisposing factors include knowledge, attitudes, beliefs, values, perceptions, and motivations that predispose individuals to engage in specific behaviors. Enabling factors are those that facilitate a person’s ability to obtain health-related services or engage in healthy behavior, and focus largely on the skills needed to engage in the behavior and the availability of resources (eg, cost factors and resource factors). Reinforcing factors aim to provide supports, which are necessary for individuals to maintain healthy behaviors, and can include social networks and positive reinforcement based on self-monitoring data and feedback information [17]. These three factors direct us to group mobile apps into three categories based on whether they focus on predisposing, enabling, or reinforcing factors that aim to affect behavior change [8]. A summary of mobile apps in relation to PPM model is provided in Table 1.

After we designed and constructed the classification scheme, we determined the face validity of this scheme by interviewing 11 people who had not been involved in the development of the study in any way; that is, they had participated in neither the construction of the classification scheme nor the content analysis [24]. They all agreed that this scheme accurately reflected the nature of this application.

Prior to conducting the reliability analysis, two researchers and a research assistant (none of whom had been involved in the development of the study’s classification scheme, content analysis, or determination of face validity) all received systematic training in the coding of the apps. Training sessions were held three times a week for 2 weeks, with each session lasting 50 minutes. The core elements of that training were to review the distinctions for coding apps into appropriate categories (eg, predisposing, enabling, and reinforcing factors of the PPM model; prevention and management for health care process; and physical versus health conditions for health conditions) and related procedures regarding study sample entry and app coding. The two researchers independently coded the study apps based on manufacturers’ written descriptions of each app, to test the scheme for its ability to categorize all the potential apps in the health and fitness categories. In accordance with our classification scheme, each mobile app was coded based on the three dimensions discussed above.

**Table 1:** Mobile app functions in relation to PPM.

Mobile apps	Features	Core values
Predisposing apps	Provide health information to impact health perceptions, health beliefs, values, or attitudes toward behavior change (eg, providing information on risks of diabetes, including obesity).	Promoting correct perceptions about the relation between lifestyle behaviors and the development of chronic disease, as well as about the value of self-management in delaying onset of disease progression and functional loss.
Enabling apps	Teach a skill (eg, how to monitor blood pressure), provide a service; record/track behaviors (eg, an app that records blood pressure values).	Providing useful and direct help to enable people to do something.
Reinforcing apps	Interface with online community using a social network site; provide encouragement from trainers/coaches; evaluate users’ self-monitoring (eg, give an evaluation based on blood pressure values).	Strengthening behaviors through interactions, typically positive feedback; emphasis on support through interaction with users.

### Sample

A sample of existing mobile apps, currently available online from the “Health & Fitness” category in the Apple iTunes App Store (ordered by released time or by alphabet), was selected to test our classification scheme. There were more than 23,000 mobile apps in the “Health & Fitness” category available to download, so we decided to randomly choose apps released during certain time periods. Because the US Food and Drug Administration (FDA) released draft guidance for the development of mobile health apps in July 2011 [25], we included time periods before and after their release date to investigate its potential influence on mobile application development. Further, to analyze the change of year-on-year growth in the number of apps, we included apps released in August 2012 as well. As a result, the final study sample consisted of apps released in June 2011, August 2011, and August 2012, and the written description of each app was accessed and viewed.

**Figure 2:**
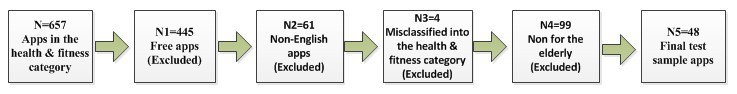
Selection of apps sample in August 2012.

**Figure 3:**
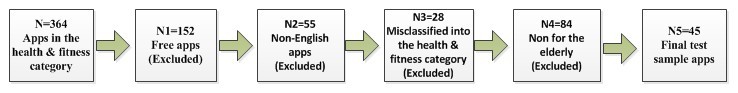
Selection of apps sample in August 2011.

**Figure 4:**
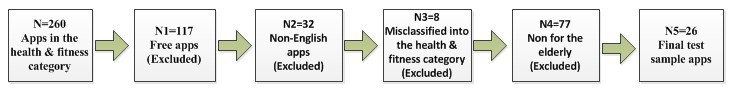
Selection of apps sample in June 2011.

Sample selection was based on the following inclusion and exclusion criteria. Inclusion criteria included: (1) apps that one had to pay for, as free apps often became upgraded apps into paid ones, leading to the possible double coding of the same app (eg, Vital mHealth released two versions of the mobile app “PainMonitoring”, a free version with limited functionality and a paid version that recorded and monitored a user’s pain level); and (2) apps whose written descriptions were in English for the purpose of global access and use. Exclusion criteria included: (1) apps misclassified and misplaced under the “Fitness and Health” category, as determined by the manufacturers’ descriptions of function not being associated with health or health care, such as bread maker timer assistant and journey scene records; and (2) apps that did not specifically relate to health or health care of older adults (eg, apps for baby health-nursing, apps for disseminating pregnancy prevention, and birth control techniques). Apps with explicit age information and/or illustrations of older adults as their target population directly helped us to identify apps for older adults. For those apps without this information, there is typically a description of the targeted health condition and functional status for the app. Knowing the health condition being targeted, as well as the functional status, enabled us to identify health-related apps for elders with specific health conditions and levels of functional status. In sum, we relied on the three types of data (ie, age information/target population, type of health condition, and functional status) to identify health and fitness apps that were not relevant for older adults. Taking August 2012 as an example, on the basis of these inclusion/exclusion criteria, there were 657 apps in the health and fitness category, with 445/657 (67.7%) free apps, 61/657 (9.3%) non-English apps, 4/657 (0.6%) misclassified apps, and 99/657 (15.1%) apps not applicable to older adults. Thus, the final study sample for August 2012 consisted of 48/657 (7.3%) apps that could be employed for older adults’ health and health care (Figure 2). App samples selected from August 2011 and June 2011 are represented in Figures 3 and 4, respectively.

### Measurement

Each app was coded according to the three dimensions in the classification scheme (see Multimedia Appendix 1). First, apps were coded in terms of health conditions, and could be coded for physical health, mental health, or both. Second, apps were coded in terms of health processes, and could be coded for management of disease, prevention of disease, or both. Third, apps were coded into the three categories of the PPM model [16,17].

### Analysis

To check the reliability of the coding scheme and justify the validity of our scheme, inter-rater reliability was conducted by two researchers (not involved in any aspect of the study), who independently coded the app data [26]. There was also a third evaluator (a research assistant who had not been involved in any aspect of the study), who was called in when there was disagreement as to the coding of the apps. We chose the entire 119 sample of apps for the purpose of validation. There was agreement (an initial level of concordance) for 114/119 (95.8%) apps, and disagreement for 5/119 (4.2%) apps. The five apps on which there was initial disagreement between coders were discussed, and when they could not arrive at consensus, the third research assistant was asked to join them until they reached consensus. Consensus was reached on these five apps as well, resulting in agreement on the coding for all 119 apps.

We were also able to develop specific combinations from the three axes of the classification scheme to provide a more refined description of the apps (eg, identifying the ratio of physical and mental apps for prevention), focusing on enabling factors.

## Results

### Study Sample

We proposed a classification scheme for the classification of existing mobile apps on the market. By using our scheme, we coded all the study sample mobile apps (Tables 2 and 3).

In the process of study sample selection, there were 379 health-related apps, and about 31.4% (119/379) of them, relevant to older adults, were chosen in the study sample, which consisted of 48/119 (40.3%) from August 2012, and 45/119 (37.8%) and 26/119 (21.8%) from August 2011 and June 2011, respectively.

### Coding Results

In August 2012, most apps were associated with physical health conditions (31/48, 64.6%), approximately two times that of mental health-related apps (17/48, 35.4%). In terms of prevention and management, apps targeted at management of disease were substantially more common than those for prevention (44/48, 91.7% vs. 6/48, 12.5%). Enabling apps took the dominant position in the PPM model (45/48, 93.8% vs. 4/48, 8.33% vs. 4/48, 8.33% for enabling, predisposing, and reinforcing, respectively), and there were few apps combining features of predisposing, enabling, and reinforcing factors. In fact, there were no such mobile apps in August 2011 or August 2012. As for physical health, apps for prevention were 16.1% (5/31), with 3.2% (1/31) apps for predisposing, 16.1% (5/31) apps for enabling, and 6.5% (2/31) apps for reinforcing. There were 64.6% (31/48) apps for management, with 3.2% (1/31), 100% (31/31), and 12.9% (4/31) apps associated with predisposing, enabling, and reinforcing factors, respectively. With respect to mental health, apps for prevention were 5.9% (1/17) and 94.1% (16/17) for management. Physical-related apps for both prevention and management were 16.1% (5/31), while mental-related apps for both prevention and management were just 5.9% (1/17).

Considering the study sample from August 2011, 64.4% (29/45) of apps were for physical health; the majority of apps (41/45, 91.1%) were used for management of disease rather than prevention of disease; enabling apps accounted for 95.6% (43/45), while predisposing and reinforcing apps were 8.9% (4/45) and 17.8% (8/45), respectively. As for physical health, 27.6% (8/29) of apps were for prevention and 93.1% (27/29) were for management, while in terms of mental health, 23.8% (5/21) of apps were for prevention and 90.5% (19/21) were for management.

Coding results of apps from June 2011 showed that 80.8% (21/26) of apps were related to physical health conditions, more than two times than that of mental health-related conditions (9/26, 34.6%). Apps for management were 92.3% (24/26) compared with 7.7% (2/26) for prevention; enabling apps were far more common than predisposing apps or reinforcing apps. In terms of physical health, 4.8% (1/21) of apps were for prevention and 95.2% (20/21) of apps were for management; and in terms of mental health, 11.1% (1/9) of apps were for prevention and 88.9% (8/9) apps were for management.

**Table 2:** Study sample (1) coding results.

Coding scheme	June 2011	August 2011	August 2012
Study sample	26	45	48
Physical-related apps, n (%)	21/26 (80.8)	29/45 (64.4)	31/48 (64.6)
Mental-related apps, n (%)	9/26 (34.6)	21/45 (46.7)	17/48 (35.4)
Prevention-related apps, n (%)	2/26 (7.7)	7/45 (15.6)	6/48 (12.5)
Management-related apps, n (%)	24/26 (92.3)	41/45 (91.1)	44/48 (91.7)
Predisposing-related apps, n (%)	2/26 (7.7)	4/45 (8.9)	4/48 (8.3)
Enabling-related apps, n (%)	26/26 (100)	43/45 (95.6)	45/48 (93.8)
Reinforcing-related apps, n (%)	8/26 (30.7)	8/45 (17.8)	4/48 (8.3)
Predisposing-enabling-reinforcing apps, n (%)	2/26 (7.7)	0/45 (0.0)	0/48 (0.0)

**Table 3:** Study sample (2) coding results.

Coding scheme	June 2011	August 2011	August 2012
Physical-prevention-predisposing, n (%)	0/21 (0.0)	1/29 (3.4)	1/31 (3.2)
Physical-prevention-enabling, n (%)	1/21 (4.8)	5/29 (17.2)	5/31 (16.1)
Physical-prevention-reinforcing, n (%)	0/21 (0.0)	2/29 (6.9)	2/31 (6.5)
Physical-management-predisposing, n (%)	2/21 (9.5)	1/29 (3.4)	1/31 (3.2)
Physical-management-enabling, n (%)	20/21 (95.2)	25/29 (86.2)	31/31 (100)
Physical-management-reinforcing, n (%)	8/21 (38.1)	6/29 (20.7)	4/31 (12.9)
Mental-prevention-predisposing, n (%)	0/9 (0.0)	0/21 (0.0)	0/17 (0.0)
Mental-prevention-enabling, n (%)	1/9 (11.1)	4/21 (19.0)	2/17 (11.8)
Mental-prevention-reinforcing, n (%)	0/9 (0.0)	1/21 (4.8)	1/17 (5.9)
Mental-management-predisposing, n (%)	0/9 (0.0)	1/21 (4.8)	3/17 (17.6)
Mental-management-enabling, n (%)	8/9 (88.9)	19/21 (90.5)	16/17 (94.1)
Mental-management-reinforcing, n (%)	0/9 (0.0)	3/21 (14.3)	2/17 (11.8)

## Discussion

### Principal Findings

In essence, our proposed scheme was a ternary relation group, represented by “HHP=<Health conditions, Health care process, PPM model>” with the capacity to identify mobile apps for disease prevention/management of physical and/or mental health conditions according to the three factors, and to classify apps with these three dimensions (Figure 1). The scheme provides a comprehensive way to classify existing mobile apps by taking into consideration of three factors, all of which are both relevant to older adults to enable them to choose the most appropriate apps to meet their needs, health care needs, and relevant to developers interested in the development of mobile apps for health care.

The main effects of the scheme (the domain between the y-axis and z-axis, projected on the yoz plane) reflected a binary relation, that is “HP1=<Health care process, PPM model>”. This scheme did not include physical/mental health conditions, and was limited to an examination of health care processes (disease prevention/management) and PPM. Every element in this binary relation indicated what kind of apps could be used for prevention and/or management of disease. This enabled us to understand the general relationship between health care processes and the PPM model, for example, what is the proportion of prevention apps targeted at predisposing, enabling, and reinforcing factors, respectively, and what is the ratio of prevention apps and management apps that are associated with enabling factors.

Likewise, from the depression view of our scheme (Figure 1), it was mapped to the xoy plane (between the x-axis and y-axis) and we denoted it by “HH=<Health conditions, Health care process>”, expressing the relationship between health conditions and health care process, which allowed an examination of which prevention and/or management apps were appropriate to physical and/or mental health conditions. This ability offers the potential to facilitate user access to the most appropriate apps to satisfy their needs in taking prevention and/or management measurements for health conditions.

If examined from the side view, the scheme was mapped to the xoz plane (between the x-axis and z-axis), denoting a binary relation between health conditions and PPM model, and we expressed it by “HP2=<Health conditions, PPM model>”, which allowed us to identify apps that deal with a certain health/mental health condition, by whether they provide predisposing, enabling, or reinforcing benefits. This is important, as behavior change theory posits that individuals benefit from specific factors in the PPM model in terms of preventing or managing their physical health and/or mental health conditions.

With the coding results of our study sample from June 2011, August 2011, and August 2012, we have the following observations and conclusions. First, compared with apps released in June 2011 (260), the number of apps in August 2011 (364) increased by 40.0%; and compared with August 2011 (364), the number of apps released in August 2012 (657) increased by 80.0% and increased 2.5 times compared with June 2011 (260). In addition, excluding free, misclassified, and non-English apps, apps applicable for older adults accounted for about one third of the entire study sample: 25.2% (26/103) for June 2011, 34.9% (45/129) for August 2011, and 32.6% (48/147) for August 2012. These data demonstrate that apps for older adults had an increase after the FDA released draft guidance to regulate their manufacture and use. It might be that app developers believed that more consumers would accept and use apps after the promulgation of FDA draft guidance related to app performance and safety, and that, as a result, they thought the timing was right for the development of new mobile apps for older adults.

Second, in terms of the PPM model, the most commonly coded apps in our study were enabling apps. Apps including all three factors of the PPM model (ie, predisposing, enabling, and reinforcing factors) were very few, supporting the findings of West et al [8]. This enhances the validity of our findings, as even though they are only related to apps targeting older adults, they arrive at a similar conclusion as West et al who included a larger range of apps appropriate for all age groups [8].

Third, mobile apps users and developers were able to obtain deeper and more comprehensive information about existing mobile applications in choosing and developing apps by using our classification scheme. Specially, the main motivation for the development of this scheme and its potential uses can be summarized as follows: (1) if combining the y-axis and z-axis, we obtained the relationship between health care processes and PPM, providing information on the relative distribution of predisposing, enabling, and reinforcing apps for prevention and/or management apps; (2) if considering the x-axis and z-axis together, we obtained relevant information about health conditions and PPM, providing information about the relative distribution of predisposing, enabling, and reinforcing apps for physical and/or mental health conditions; and (3) if examining both the x-axis and y-axis, we obtain the binary relation between health conditions and health care processes and further identify the relative distribution of prevention and/or management apps for a specific physical/mental health condition. In summary, the proposed classification scheme provides a more comprehensive way to analyze a mobile application from the view of health conditions (ie, physical and/or mental health conditions), health care processes (choosing which kind of health process, prevention or management of disease), and PPM (which factors influence behavior change).

Both older adult app users and mobile app developers could benefit from such a classification scheme. For older adult app users, our classification scheme can facilitate the choice of appropriate mobile apps according to their actual needs for prevention and/or management, physical and/or mental health conditions, and which kind of factor they are in need of for behavior change. For example, an older person, hoping to improve his/her physical health conditions with the second phase of behavior change, would receive a list of mobile apps related to physical health improvement, which provide enabling factors, such as tools to track physical activity level and diet.

For mobile app developers, our classification scheme has the capacity to reveal the relative distributions of each type of mobile apps. Our classification scheme does not just present developers simple statistical information about the ratio between prevention apps and management apps, or the relative number of physical health-related apps and mental health-related apps and the distributions of apps in predisposing, enabling, and reinforcing categories. Our scheme also provides valuable and concrete information when we combine different components of our scheme to classify existing mobile apps. For example, app developers would be able to identify the current availability of apps with predisposing factors for the prevention of mental conditions as well as the availability of apps with reinforcing factors for the prevention of mental conditions. Thus, our classification scheme has great potential in providing insightful and dynamic information to mobile app developers and in helping developers make informed decisions about the development of future mobile apps for the prevention and management of disease by using the proposed classification scheme to categorize all existing mobile apps on the market.

###  Limitations

This study presented one of the first of its kind to construct a classification scheme for the categorization of paid mobile apps publicly available in the Apple iTunes App Store in a comprehensive way by considering the following three dimensions: the PPM model, health care process (prevention and management), and health care conditions (physical health and mental health). However, there are several limitations to the study. First, in constructing our classification scheme, we adopted a generalized, rather than a finer-grained one. For example, health conditions were grouped simply into physical health conditions and mental health conditions, but not classified more specifically as those listed in the HECAT, such as alcohol misuse, tobacco and other drug use, healthy eating, mental and emotional health, personal health and wellness, physical activity, safety, sexual health, and violence prevention [18]. This limited us from providing older adults with specific information regarding specific health conditions in which they might be interested. It also precluded us from providing mobile app developers with more specific information regarding the number of apps in each domain listed in HECAT and correspondingly, which kind of more specific apps were less available in the market. Instead, we provided a more general impression on app distribution in terms of physical health conditions and mental health conditions. Second, as indicated above, we chose to consider those apps that would have potential relevance for older adults, and not people of all ages. Our scheme has the capacity to categorize mobile apps for other age groups, such as babies, teenagers, and young adults, by providing target group relevant mobile apps as study samples. However, this was not the purpose of our study. Future research could use the age group of user as a covariate to our classification scheme, utilizing all existing mobile apps, which would assist other age group users in the choice of mobile apps that are appropriate to their specific health concerns. Third, while the goals of the mobile apps we included were to support older adults to prevent disease, maintain or improve their health and fitness, and manage their physical and mental health conditions, the classification scheme is unable to incorporate users’ appraisals of the mobile apps included in the analysis. As a result, we have no idea about how end users (eg, older adults and their family caregivers) evaluated their effectiveness, usability, and/or acceptability, which could provide valuable information to app designers and developers, as well as to other older consumers looking for specific apps.

### Conclusions

A number of mobile apps are available on the market, and these apps offer the promise of not only enhancing the self-management of disease among older adults, but also promoting health behaviors that could potentially prevent or delay the onset of disease. There are mobile apps for enhanced disease management for a number of chronic diseases, including diabetes, hypertension, and stroke; health care information education dissemination; and for collecting individual information and storing data in a center server accessible to professional physicians and/or family caregivers and transmitting the data to professional physicians and/or family caregivers.

For the purpose of acquiring a comprehensive view of available apps on the market, we put forward and constructed a classification scheme for the categorization of mobile apps by considering the following three dimensions: (1) the PPM (ie, predisposing, enabling, and reinforcing factors), (2) health care process (ie, prevention vs. management of disease); and (3) health conditions (ie, physical vs. mental health). Using our classification scheme, a content analysis of mobile apps was conducted to classify the study sample apps in the “Health and Fitness” category publicly available in the iTunes App Store. Face validity and test-retest reliability were demonstrated. We believe that the proposed classification scheme can potentially help older adult app users more easily locate appropriate apps for their specific needs. We also believe that the proposed classification system can potentially help app developers understand the existing distribution of mobile apps and provide important information to identify current gaps in available mobile applications, so that they can make better-informed decisions about the development of future mobile applications for the prevention and management of disease in late life. Future work could expand the classification scheme to all mobile apps, and include age as a covariate. In addition, future work would benefit by taking user appraisal into account when classifying mobile apps, enabling the quantification of users’ subjective evaluation of apps.

## Multimedia Appendix 1

App coding form.

## Abbreviations

eCAALYX: Enhanced Complete Ambient Assisted Living Experiment
FDA: US Food and Drug Administration
HECAT: Health Education Curriculum Analysis Tool
PC: personal computer
PDA: personal digital assistant
PPM: Precede-Proceed Model
