# Supplementary material for: A Classification Scheme for Analyzing Mobile Apps Used to Prevent and Manage Disease in Late Life
Source: JMIR Mhealth Uhealth. 2014 Feb 17;2(1):e6. doi: 10.2196/mhealth.2877 (PMC4114434; doi:10.2196/mhealth.2877)
Supplement: Supplementary file 1 [file mhealth_v2i1e6_app1.pdf]

Multimedia Appendix 1: App coding form

- Could this app be used for health (i.e., misclassified or not)?

1. Yes
2. No (if No, stop coding this app)

- Could this app be used for elderly health or healthcare? (i.e., check no if it is for baby' health, pregnant women, etc.)

    1. Yes
    2. No (if No, stop coding this app)

- Is this app for physical or mental health conditions or both?

1. Physical conditions
2. Mental conditions
3. Both

- Is this app for prevention or management of disease, or for both (i.e., prevention is to prevent one from disease or illness, physical or mental; meanwhile, management focusing on what to do when suffering from disease or illness)?

1. Disease prevention
2. Disease management
3. Both

- Predisposing-factors that influence behavior (things that precede behavior and provide reasons and motivation for behavior, and they are cognitive or affective based, like attitudes, knowledge, awareness, beliefs, information, values, confidence, motivation, self-efficacy, etc.)

1. Yes
2. No

- Enabling-factors that influence behavior (Occurs at or around the same time as behavior and it facilitates or weakens behavior, such as teaching a skill, provide a service, track or record behavior)

  1. Yes
  2. No

- Reinforcing-factors that influence behavior (Interactive applications, interface with SNS, encouragement from trainers/coaches, evaluation based on self-report)

  1. Yes
  2. No

- Level of predisposing

  1. Knowledge or awareness of a health behavior/outcome
  2. Informative
  3. Beliefs, values, attitudes
  4. Confidence, motivation

- Level of enabling

  5. Teaching a skill
  6. Providing service (sells something, sign-up for events, facilitates engaging in a behavior)
  7. Tracking/recording behavior

- Level of reinforcing

  8. Interfacing with SNS (encouragement from social support)
  9. Encouragement-trainer support, coach
  10. Evaluation based on self-report
